# Striatal Chloride Dysregulation and Impaired GABAergic Signaling Due to Cation-Chloride Cotransporter Dysfunction in Huntington’s Disease

**DOI:** 10.3389/fncel.2021.817013

**Published:** 2022-01-14

**Authors:** Melissa Serranilla, Melanie A. Woodin

**Affiliations:** Department of Cell and Systems Biology, University of Toronto, Toronto, ON, Canada

**Keywords:** KCC2, chloride regulation, GABA, synaptic inhibition, striatum, Huntington’s disease

## Abstract

Intracellular chloride (Cl^–^) levels in mature neurons must be tightly regulated for the maintenance of fast synaptic inhibition. In the mature central nervous system (CNS), synaptic inhibition is primarily mediated by gamma-amino butyric acid (GABA), which binds to Cl^–^ permeable GABA_A_ receptors (GABA_A_Rs). The intracellular Cl^–^ concentration is primarily maintained by the antagonistic actions of two cation-chloride cotransporters (CCCs): Cl^–^-importing Na^+^-K^+^-Cl^–^ co-transporter-1 (NKCC1) and Cl^–^ -exporting K^+^-Cl^–^ co-transporter-2 (KCC2). In mature neurons in the healthy brain, KCC2 expression is higher than NKCC1, leading to lower levels of intracellular Cl^–^, and Cl^–^ influx upon GABA_A_R activation. However, in neurons of the immature brain or in neurological disorders such as epilepsy and traumatic brain injury, impaired KCC2 function and/or enhanced NKCC1 expression lead to intracellular Cl^–^ accumulation and GABA-mediated excitation. In Huntington’s disease (HD), KCC2- and NKCC1-mediated Cl^–^-regulation are also altered, which leads to GABA-mediated excitation and contributes to the development of cognitive and motor impairments. This review summarizes the role of Cl^–^ (dys)regulation in the healthy and HD brain, with a focus on the basal ganglia (BG) circuitry and CCCs as potential therapeutic targets in the treatment of HD.

## Introduction

Huntington’s disease (HD) is an inherited neurodegenerative disorder characterized by involuntary choreatic movements, cognitive disturbances, and mood disorders (Ghosh and Tabrizi, 2018). HD is caused by a triple cytosine-adenine-guanine (CAG) repeat expansion in the gene encoding for the ubiquitously expressed Huntingtin (Htt) protein (The Huntington’s Disease Collaborative Research Group, 1993), with the CAG repeat length corresponding to the age of onset and disease severity (Snell et al., 1993). HD is primarily characterized by progressive motor incoordination as earlier stages of the disease include involuntary movement (chorea), while late stages are characterized by progressive hypokinesia and bradykinesia (Huntington, 1872). These biphasic changes that occur during disease progression result from differential susceptibility in the direct and indirect pathways of basal ganglia (BG) circuitry (Albin et al., 1992). The direct and indirect pathways exert opposing effects on BG output, activation of the direct pathway promotes movement while activation of the indirect pathway inhibits unwanted movements (Alexander and Crutcher, 1990). In HD, gamma-amino butyric acid (GABA)-releasing medium spiny neurons (MSNs) of the indirect pathway, which primarily express the D2 dopamine receptor (D2-MSNs) demonstrate an enhanced susceptibility to neurodegeneration, leading to the development of chorea (Albin et al., 1992). At late stages of the disease, MSNs of the direct pathway which primarily express the D1 dopamine receptor (D1-MSNs) also degenerate leading to rigidity and hypokinesia (Thompson et al., 1988).

Impaired GABAergic signaling directly contributes to the cognitive and motor deficits associated with HD, and results in part from alterations in two cation-chloride cotransporters (CCCs): Na^+^-K^+^-Cl^–^ co-transporter-1 (NKCC1) and K^+^-Cl^–^ co-transporter-2 (KCC2). In mouse models of HD, NKCC1 and KCC2 are altered in the hippocampus and striatum, which results in weakened inhibition and paradoxical excitatory actions of GABA (Dargaei et al., 2018; Hsu et al., 2019). In this review, we highlight Cl^–^ regulation by CCCs and its impact on GABA signaling to explore the potential mechanisms underlying CCC dysfunction in HD. We start by reviewing: HD, CCCs, Cl^–^ dynamics and GABAergic transmission in the BG of healthy and HD brains. Lastly, we consider why existing drugs targeting GABA signaling may have failed in treating HD (Shoulson et al., 1977; Foster et al., 1983) and how targeting CCCs may prove to be a better alternative for restoring inhibitory function in this devastating neurodegenerative disease.

## Huntington’s Disease

### Overview

The first description of HD dates as early as 1842, but it was not until 1872, that it became known as Huntington’s chorea, which refers to the rapid and irregular movements first observed (Huntington, 1872). Motor symptoms begin as hyperkinetic movements which decrease as patients develop bradykinesia and rigidity, however, in later stages HD patients experience severe hypokinesia and an akinetic state (Sturrock and Leavitt, 2010). The psychiatric symptoms associated with HD are highly variable and precede motor symptoms by ∼10-15 years, making it difficult to diagnose HD without genetic tests.

**FIGURE 1:**
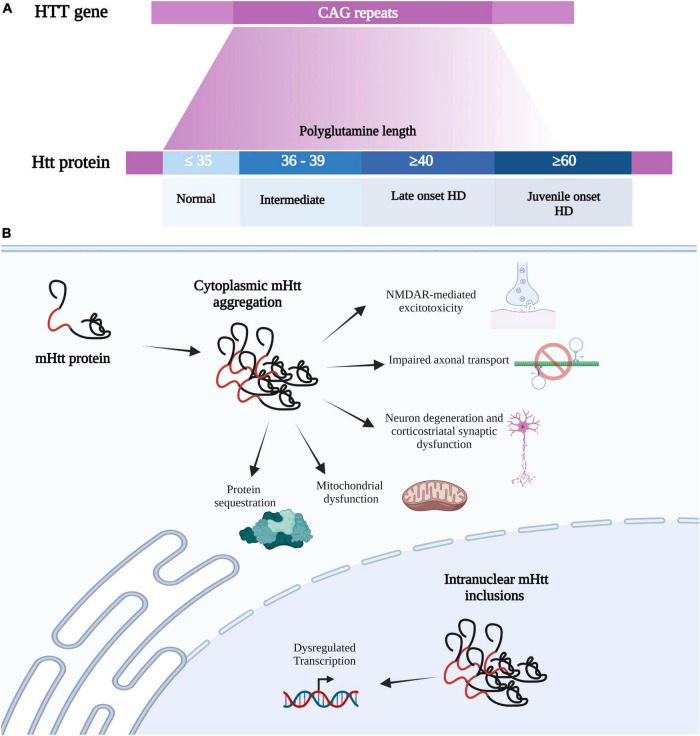
Huntington’s disease mutation and HD-associated cellular impairments. **(A)** HD is a caused by a mutation in the *HTT* gene on chromosome 4, which consists of CAG (cytosine-adenine-guanine) trinucleotide repeats which encode for a glutamine. The *HTT* mutation is an expanded CAG, where normal individuals contain 35 or fewer CAG repeats while HD carriers contain 36 or more. CAG repeat length is inversely correlated with disease onset and symptom severity, with over 60 CAG repeats leading to juvenile-onset HD. The CAG repeat expansion gives rise to an aberrantly long polyglutamine stretch in the mutant huntingtin protein (mHtt), causing the protein to misfold and obtain toxic properties. **(B)** mHtt can form insoluble aggregates in the cytosol and nucleus to produce various toxic effects. As normal Htt has multiple binding partners, mHTT can sequester these proteins into these mHtt aggregates. mHtt aggregation also causes mitochondrial dysfunction, neuron degeneration (primarily at corticostriatal synapses), impairs axonal transport, and mediates glutamate toxicity. mHtt can also be translocated into the nucleus to form Intranuclear mHtt inclusion, which lead to dysregulation transcription. Created with BioRender.com.

Huntington’s disease is caused by a mutation in the gene (*HTT*) encoding the protein huntingtin (Htt) on chromosome 4. The *HTT* mutation is a repeat expansion of cytosine-adenine-guanine (CAG) which encodes for the amino acid glutamine in the first exon (The Huntington’s Disease Collaborative Research Group, 1993). CAG repeat length is inversely correlated with age of disease onset and symptom severity, with healthy individuals having 35 or fewer CAG repeats while HD gene carriers contain 36 or more CAG repeats. Over 60 CAG repeats leads to the development of juvenile-onset HD, which occurs in 5-10% of HD patients and is associated with increased severity (Andrew et al., 1993; Figure 1A). This CAG repeat expansion gives rise to an aberrantly long polyglutamine stretch in the mutant huntingtin (mHtt), causing the protein to misfold and to obtain toxic properties (Alexander and Crutcher, 1990) (Figure 1B). Despite the ubiquitous expression of mHtt throughout the brain of HD patients, neurodegeneration is specific to certain brain regions such as the striatum, cerebral cortex, and hippocampus (Reiner et al., 1988; Spargo et al., 1993).

Current therapeutic approaches are limited by the late detection of the disease, which primarily relies on the presence of motor impairments, although recently more sensitive methods have been developed to evaluate subtle disturbances in HD (Paulsen et al., 2008). Without direct targeting of mHtt, the current treatments are limited to targeting and mitigating symptoms, which include speech and physical therapies (Dickey and La Spada, 2018) and modulators of dopaminergic signaling such as tetrabenazine to treat motor symptoms (Jankovic and Clarence-Smith, 2011). While the use of Antisense Oligonucleotides (ASOs), RNA Interference, RNA Splicing Modification and DNA Repair Proteins (Wiggins and Feigin, 2021) hold some promise, clinical trials do not include premanifest HD patients or those with the early-onset juvenile HD (Wiggins and Feigin, 2021), further impeding the development of HD therapeutic strategies. As we discuss below, CCCs have only recently emerged as a potential therapeutic target and have proven to be beneficial in both presymptomatic and symptomatic HD mice (Dargaei et al., 2018; Hsu et al., 2019), providing a novel strategy to combat this devastating disease.

### Interactors of Huntingtin and K^+^-Cl^–^ Co-transporter-2 Are Altered in Huntington’s Disease

Although the HD mutation has been well-characterized, the mechanism by which mHtt protein leads to a specific pattern of synaptic dysfunction remain elusive. Normal Htt is a multidomain protein with no sequence homology with other proteins, thus efforts to find its exact functions have relied on examining interacting proteins (Harjes and Wanker, 2003). Htt has over 200 protein interactors that have functions in: cellular dynamics, metabolism, protein turnover, and gene expression (Saudou and Humbert, 2016), which suggests that Htt may be a molecular scaffold that tethers multiple partners into complexes necessary for various signaling processes. With the many functions of Htt and large number of interacting proteins, we highlight only those relevant to CCC activity and inhibitory synaptic transmission. For example, an important regulator of KCC2 transcription is brain-derived neurotrophic factor (BDNF), an essential neurotrophin for the proper functioning of cortico-striatal synapses (Nakao et al., 1995). BDNF production and trafficking in the striatum are regulated by Htt and are well studied in HD (Zuccato et al., 2003). BDNF transcripts contain DNA repressor elements (RE1), also referred to as neuron-restrictive silencer element (NRSE) sequences, which is recognized by the transcriptional silencer RE-1 silencing transcription factor (REST), also known as neuronal restrictive silencing factor, (NRSF) (Maue et al., 1990). Normal Htt promotes *Bdnf* transcription by sequestering REST/NRSF in the cytoplasm, thus preventing it from forming the nuclear co-repressor complex at the RE/NRSE nuclear site, thereby allowing BDNF transcription to take place. In contrast, mHtt allows entry of REST/NRSF into the nucleus, thereby reducing *bdnf* transcription (Zuccato et al., 2003).

Another important regulator of KCC2 activity that is altered in HD is protein kinase C and casein kinase II substrate in neurons (PACSIN1) (Mahadevan et al., 2017). Htt interacts with PACSIN1, which regulates post-synaptic expression of N-methyl-D-aspartate receptors (NMDARs) by removing the immature GluN3A subunits necessary for the incorporation of more mature subunits in the membrane (Modregger et al., 2002). This prevents premature synapse plasticity and provides stability during early stages of postnatal brain development (Pérez-Otaño et al., 2006). In HD, mHtt sequesters PACSIN1, causing accumulation of GluN3A-containing NMDARs at the surface of striatal neurons leading to synapse destabilization and synaptic degeneration in HD (Marco et al., 2013).

### Huntington’s Disease Impacts Neurodevelopment

Huntington’s disease is largely considered a neurodegenerative disease, with very little impact on development due to late onset of symptoms and the ability of mHtt expression to rescue embryonic lethality in *HTT*-null mice (Zeitlin et al., 1995; Leavitt et al., 2001). However, the HD mutation is now known to produce neurodevelopmental defects, suggesting that the disease is much more complex (Barnat et al., 2017; Barnat et al., 2020; Capizzi et al., 2021). Htt is required for the multipolar-bipolar transition and migration of projection neurons, and in HD newborn cortical neural migration is disrupted (Barnat et al., 2017). At 13-week gestation in the fetal cortex, junctional complex proteins (in addition to mHtt) are mislocalized and neural progenitor cell polarity and differentiation are defective, leading to premature entry of neural progenitors entering lineage specification (Barnat et al., 2017), which has led to the suggestion that Htt maintains epithelial cell polarity throughout the body. In addition, a recent study reported that layer II/III neurons exhibit defects in microtubule bundling leading to limited axonal growth during development (Capizzi et al., 2021). The disorganization of microtubules is due to the downregulation of nuclear mitotic apparatus protein 1 (NUMA1), which plays a role in the proper formation and organization of mitotic spindles during cell division (Kiyomitsu and Boerner, 2021). Due to the limited axonal growth, fewer axons cross the corpus callosum, which normally connect the two hemispheres. It is now evident that the HD mutation disrupts fundamental processes during neurodevelopment such as neurogenesis, neural migration, and axonal growth.

Although this review focuses on CCC dysfunction in mature neurons, the neurodevelopmental defects in HD and the importance of GABA signaling during the development of neural networks (Ben-Ari, 2002) presents the possibility that in HD, specific neurons may have failed to undergo the necessary “developmental switch” in GABA polarity, which we describe below.

## Cation-Chloride Co-Transporters, Chloride Regulation and Synaptic Inhibition

### Overview

**FIGURE 2:**
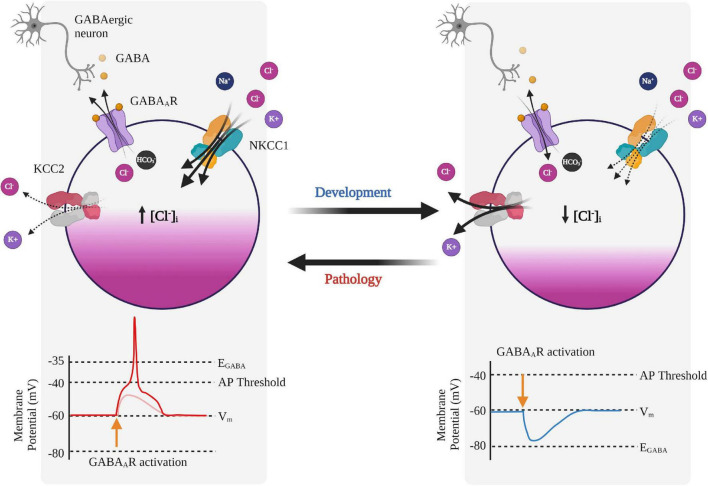
Neuronal Cl^–^ regulation and its effects on GABA_A_ receptor signaling during development and pathology. GABA_A_ receptor (GABA_A_R) signaling is developmentally regulated and switches from depolarizing to hyperpolarizing. In immature neurons, the GABA response is depolarizing due to relatively higher expression of NKCC1 compared to KCC2, resulting in Cl^–^ efflux upon GABA_A_R activation, and subsequent depolarization of the membrane potential. In mature neurons, expression of KCC2 increases leading to Cl^–^ influx upon GABA_A_R activation and a hyperpolarizing response of GABA. In some pathologies, KCC2 activity is reduced and can promote reversion back to a phenotypically immature state and a depolarizing response of GABA, which can also lead to GABA-mediated excitation. Created with BioRender.com.

Gamma-amino butyric acid is the main inhibitory neurotransmitter in the mature central nervous system (CNS), which mediates fast GABAergic inhibition by binding to GABA_A_Rs. GABA_A_Rs are permeable to Cl^–^ and HCO_3_^–^ and therefore the reversal potential of GABA_A_R currents (E_GABA_) is determined by the equilibrium potentials of Cl^–^ (E_Cl_) and HCO_3_^–^ (E_HCO3_) (Figure 2; Kaila et al., 1989; Kaila, 1994). The equilibrium potential for a particular ion is the membrane potential at which there is no net flow of that ion and can be calculated from the Nernst equation (if the intra- and extracellular concentrations are known). In healthy mature neurons, E_Cl_ is usually slightly hyperpolarized with respect to the resting membrane potential, while E_HCO3_ is depolarized, ranging from −40 mV to −20 mV (Kaila, 1994). Thus, the driving force (DF), which is the difference between the membrane potential and the equilibrium potential (DF = V_m_ – E_ion_), is greater for HCO_3_^–^ than it is for Cl^–^ when the neuron is at rest.

The reversal potential of GABA is the membrane potential at which there is no net current upon receptor activation and is a combination of E_Cl_ and E_HCO3_. E_GABA_ can be calculated from the Goldman–Hodgkin–Katz equation, which requires knowledge of the intra- and extracellular concentrations of Cl^–^ and HCO_3_^–^ and their conductances. Because GABA_A_Rs are significantly more permeable to Cl^–^ than HCO_3_^–^ (Kaila et al., 1989; Fatima-Shad and Barry, 1993), E_GABA_ sits much closer to E_Cl_ than to E_HCO3_, and thus E_GABA_ is often taken as E_Cl_ (E_GABA_ ≈ E_Cl_).

Gamma-amino butyric acidergic transmission is plastic and can undergo short- and long-term shifts in the Cl^–^ and HCO_3_^–^ gradients in postsynaptic neurons (Rivera et al., 2005; Lamsa et al., 2010; Raimondo et al., 2012). Short-term GABAergic plasticity can be induced by high-frequency GABAergic stimulation, which dissipates the driving force for Cl^–^ and reduces the efficacy of fast GABAergic inhibition (Thompson and Gähwiler, 1989; Doyon et al., 2011, 2016). This phenomenon was described more than two decades ago, as the amplitudes of GABA_A_R-mediated inhibitory postsynaptic currents (IPSCs) were aptly described as “fading” currents (Huguenard and Alger, 1986). This demonstrates that repetitive stimulation results in weakened inhibition (disinhibition) in an activity-dependent manner, which can be due to progressive depletion of the Cl^–^ driving force (referred to as ionic plasticity) and/or the sensitization of GABA_A_Rs (Rivera et al., 2005; Raimondo et al., 2012). Bearing this in mind, it’s unsurprising that feedback inhibition such as in the BG might be more susceptible to activity-dependent disinhibition which we discuss below, although this has not been experimentally demonstrated in HD.

Long-term GABAergic ionic plasticity depends upon changes in membrane expression and transport activity of KCC2 and NKCC1, as well as carbonic anhydrase isoform VII (CAVII), which regulates intracellular pH and consequently E_HCO3_ (Kaila and Voipio, 1987; Ruusuvuori et al., 2004; Rivera et al., 2005). Repetitive pre-and postsynaptic activity of hippocampal neurons decreases the strength of inhibition, due to a depolarization of E_GABA_, which results from a reduction in KCC2-mediated Cl^–^ -extrusion (Woodin et al., 2003). It is important to note that depolarizing GABA does not necessarily result in excitation as depolarizing GABA can dampen the excitatory response in target cells via shunting inhibition. Shunting inhibition via GABA_A_R activation leads to a decreased membrane resistance, which effectively increases the background conductance consequently, spatially and temporally dampening the excitatory signal (Doyon et al., 2016).

### Cation-Chloride Cotransporters Overview

As GABA signaling depletes the Cl^–^ driving force, CCCs are key players in maintaining the efficacy of synaptic inhibition. The role of CCCs was first discovered in the 1970s, when researchers discovered that erythrocytes could recover their volume upon hypertonic shrinkage through a Na^+^-K^+^ cotransport mechanism that was sensitive to furosemide, a CCC blocker (Kregenow, 1981). Two decades later, the Na^+^-K^+^ cotransport was determined to be mediated by the “electrically silent” Na-K-2Cl cotransport mechanisms of NKCC1 and NKCC2 (Hebert and Gamba, 1994). CCCs form the *SLC12* transporter family, which facilitate electroneutral transport of Cl^–^ by coupling energetically-favorable transport of Na^+^ and/or K^+^ across the plasma membrane to regulate osmolarity and water balance (Arroyo et al., 2013). CCCs are divided into two groups: Na^+^-dependent transporters such as NKCC1, NKCC2, and NCC, while the Na^+^-independent branch includes KCC1, KCC2, KCC3, and KCC4. Two other members of the *SLC* transporter family are CCC9 and CIP, whose function still remain unclear (Blaesse et al., 2009; Arroyo et al., 2013). Despite the fact that CCCs were cloned more than two decades ago, their protein structures remained largely unknown until fairly recently with the development of more sensitive methods to uncover CCC structure with higher resolution, particularly through cryogenic electron microscopy (cryo-EM) and single-particle imaging (Chew et al., 2019; Liu et al., 2019b; Yang et al., 2020; Zhang et al., 2021). These studies uncovered two Cl^–^ binding sites specifically in human KCC1 and KCC4, with one involved in ion transport while the other appears to play a more facilitative role in ensuring the former is occupied via allosteric interactions (Liu et al., 2019b). Interestingly, the *N*-terminal loop performs an auto-inhibitory function which blocks cytoplasmic entry of the translocation pore (Chi et al., 2021). Indeed, improved resolution of CCC tertiary structures will likely facilitate the much-needed uncovering of CCC-interacting peptides or regulatory molecules as potential therapeutic strategies.

### K^+^-Cl^–^ Co-transporter-2 Expression and Function

KCC2 is encoded by the *SLC12A5* gene (Sallinen et al., 2001) and unlike the other CCCs, KCC2 is constitutively active, operating under isotonic conditions due to the presence of a 15-residue C-terminal domain (Mercado et al., 2006). KCC2 is largely neuron-specific (Williams et al., 1999) and is known for its role in maintaining Cl^–^ homeostasis in adult neurons by extruding Cl^–^ against its concentration gradient driven by the energetically favorable extrusion of K^+^ (Gamba, 2005; Chamma et al., 2012). KCC2 dysfunction leads to increased intracellular Cl^–^ and reversion to a developmentally immature state with depolarizing GABA and increased neuronal excitability (Figure 2; Ben-Ari et al., 2012a). KCC2 is also considered a moonlighting protein, with roles outside of ion transport which include cell migration, dendritic formation, spine morphology and synaptogenesis mediated through interactions with the dendritic cytoskeleton (Kaila et al., 2014; Blaesse and Schmidt, 2015). The cytosolic C-terminal tail of KCC2 interacts directly with the cytoskeletal protein 4.1N to provide dendritic structural support (Li et al., 2007). Without KCC2, dendritic spine morphology is compromised, producing stubby structures that compromise the function of excitatory synapses (Li et al., 2007). Indeed, with the broad functions (independent of ion transport) of KCC2, it’s no surprise that reduced GABAergic inhibition is not the only phenotype in KCC2-related pathologies and may contribute to other symptoms in HD (Tang, 2020; Virtanen et al., 2021).

During development GABA transmission is depolarizing, which is thought to provide the main source of excitatory drive during activity-dependent formation of neuronal networks (Ben-Ari et al., 1989). However, the developmental switch in GABA polarity remains controversial (Ben-Ari et al., 2012b; Bregestovski and Bernard, 2012; Zilberter, 2016) due to discrepancies between GABA actions *in vitro* (Ben-Ari et al., 1989, 2007) and *in vivo* (Valeeva et al., 2016). The switch in the GABA-mediated response is determined by the relative surface membrane expression of KCC2 and NKCC1, although the pattern of expression seen throughout development is neuron-type and species-specific (Rivera et al., 1999; Li et al., 2002). At approximately postnatal day 15 (P15), KCC2 mRNA increases, exceeding levels of NKCC1 mRNA, leading to GABA’s hyperpolarizing response in the cortex and hippocampus of rats (Wang et al., 2002). At around P21, KCC2 protein expression stabilizes and reaches mature levels (Takayama and Inoue, 2007), reducing intracellular Cl^–^ and producing a hyperpolarizing shift in E_GABA_ (Mueller et al., 1984). The upregulation of KCC2 transcripts is regulated by various neurotrophic factors (Watanabe and Fukuda, 2015), such as BDNF and its receptor tropomyosin-related kinase B (TrkB) (Ludwig et al., 2011). Interestingly, BDNF-TrkB signaling in mature neurons produces the opposite effects, leading to the downregulation of KCC2 (Rivera et al., 2002). Given the severely altered levels of BDNF in HD and BDNF’s role in regulating KCC2 transcripts, we hypothesize that there may be a causal relationship between the reduction in BDNF and KCC2 dysfunction in HD, though the mechanisms underlying this alteration requires further investigation.

K^+^-Cl^–^ co-transporter-2 expression is largely mediated through membrane trafficking and stability in the plasma membrane, a process mainly regulated by (de)phosphorylation (Russell, 2000; Rinehart et al., 2009; Kahle et al., 2013). An important regulatory phosphorylation site is serine 940 (S940), a target of protein kinase C (PKC) (Lee et al., 2007). PKC-mediated phosphorylation of S940 increases stability in the plasma membrane by reducing lysosome-dependent cleavage and the rate of internalization from the membrane. In contrast, NMDA receptor-mediated de-phosphorylation of S940 via protein phosphatase 1 (PP1) reduces KCC2 activity by the rate of KCC2 internalization at the plasma membrane (Lee et al., 2011). Reduction of KCC2 expression can also occur through the calcium-activated protease calpain, which is mediated by MAPK phosphorylation (Puskarjov et al., 2012). KCC2 phospho-regulation can also occur through two other threonine residues, T906 and T1007. These residues are phosphorylated by the With no lysine kinase (WNK)-regulated Ste20-related proline/alanine-rich kinase (SPAK)/Oxidative stress response 1 (OSR1) kinases. Activation of the WNK-SPAK/OSR1 pathway leads to increased intracellular Cl^–^ by inhibiting KCC2 and increasing NKCC1 activity (Rinehart et al., 2009; Gagnon and Delpire, 2012; de Los Heros et al., 2014). Given that activation of the WNK-SPAK/OSR1 pathway produces antagonistic effects on KCC2 and NKCC1, it remains to be determined whether the phosphorylation status of either KCC2 and/or NKCC1 are impacted in HD.

### Na^+^-K^+^-Cl^–^ Co-transporter-1

Na^+^-K^+^-Cl^–^ co-transporter-1 is encoded by the *SLC12A2* gene and produces antagonistic effects to KCC2. NKCC1 mediates cellular Cl^–^ influx which is driven by the highly favorable Na^+^ inward direction. In addition to expression in the CNS, NKCC1 is broadly expressed such as in the salivary gland, sweat gland, lungs, and intestine (Delpire and Gagnon, 2018; Koumangoye et al., 2021), which can lead to unwanted side effects in NKCC1 drug targeting. NKCC1 activity is regulated by the WNK-SPAK kinase pathway, whereby phosphorylation by SPAK and OSR1 on the *N*-terminal activates transporter activity (Gagnon et al., 2007). Due to the high degree of flexibility, the tertiary structure and *N*-terminal domain of NKCC1 had been difficult to elucidate until recently (Chew et al., 2019). NKCC1 is sensitive to loop diuretic drugs such as furosemide and bumetanide used to lower blood pressure and a therapy for cardiovascular diseases (Chobanian et al., 2003).

## K^+^-Cl^–^ Co-Transporter-2 and Na^+^-K^+^-Cl^–^ Co-Transporter-1 and Chloride Dysregulation in Neurodegenerative and Neurodevelopmental Disorders

### Overview

Alterations in CCC expression occur in both neurodegenerative and neurodevelopmental disorders. Most commonly, in neuronal hyperexcitability, which in turn, promotes epileptic seizures (Kahle et al., 2008; Moore et al., 2017). Moreover, reduction of pathological increases in intracellular Cl^–^ used to restore GABAergic inhibition have proven to reduce seizure severity (Dzhala et al., 2008; Mazarati et al., 2009). CCC targeting has been particularly effective in clinical trials with bumetanide used to reduce intracellular Cl^–^ in autism spectrum disorders (ASDs) (Ben-Ari, 2017) such as in Rett syndrome (RTT), a disorder caused by a mutation in the transcriptional repressor Methyl CpG binding protein 2 (MeCP2) gene (Chahrour et al., 2008). In RTT, GABAergic inhibition is impaired due to reduced KCC2 function which can be rescued by overexpressing KCC2 in MeCP2-deficient neurons (Tang et al., 2016) or using KCC2 expression-enhancing compounds (KEECs) (Tang et al., 2019). Similarly, KCC2 downregulation following neuropathic pain has been well studied (Hasbargen et al., 2010; Kahle et al., 2014; Chen et al., 2018). Neuropathic pain leads to disinhibition in the spinal cord dorsal horn, which could be rescued by enhancing Cl^–^ extrusion by KCC2 in rodent models (Kahle et al., 2014; Chen et al., 2018). But what about the involvement of KCC2 and NKCC1 in neurodegenerative diseases? Below we outline the recently reported experimental evidence of CCC dysfunction in neurodegenerative diseases, such as HD.

### K^+^-Cl^–^ Co-transporter-2 and Na^+^-K^+^-Cl^–^ Co-transporter-1 and Chloride Dysregulation in the Huntington’s Disease Brain

The link between altered CCC transcripts in HD was first identified in an unbiased interactome study of the unfolded protein response (UPR) in HD (Kalathur et al., 2015). To elucidate the mechanisms underlying cell apoptosis, Kalathur et al. (2015) examined the UPR, which is activated by accumulated misfolded protein in the endoplasmic reticulum (Kalathur et al., 2015). The authors examined UPR gene expression, and discovered that *SLC12A5* (KCC2) was downregulated and *SLC12A2* (NKCC1) was upregulated. Moreover, proteomic analysis of Htt-interacting proteins ranked *SLC12A5* as one of the topmost Htt-correlated module containing Htt itself (Shirasaki et al., 2012).

Based on these powerful unbiased Htt-interactomes, the importance of GABAergic inhibition in learning and memory, and the known deficits in hippocampal-dependent memory in HD, Dargaei et al. (2018) aimed to characterize CCC function in the HD hippocampus (Dargaei et al., 2018). This was the first study to demonstrate impaired CCC function in HD leading to GABA-mediated excitation. Despite the various interactome studies linking Htt and KCC2 (Shirasaki et al., 2012; Kalathur et al., 2015; Mahadevan et al., 2017), the authors were the first to validate the interaction between KCC2 and Htt protein in a HD mouse model; they performed co-immunoprecipitation assays in the R6/2 model. R6/2 is a transgenic fragment model of HD that overexpresses exon 1 of the human *HTT* with ∼120-150 CAG repeats (Mangiarini et al., 1996) and although it represents the juvenile form of HD, it is the most well studied mouse model owing to early development of symptoms (Carter et al., 1999). To ensure that CCC alterations were consistent across multiple mouse models, they used another transgenic mouse model which expresses the full-length *HTT* with 128 repeats with a yeast artificial chromosome (YAC), called the YAC128 (Hodgson et al., 1999). Unlike the R6/2, the YAC128 display a slower progression of the disease and because of this late-onset, are considered to be one of the most clinically relevant mouse models of HD (Menalled and Chesselet, 2002). The authors demonstrated that in HD, hippocampal neurons reverted to an immature state, with increased NKCC1 expression and reduced KCC2 expression, which may underlie cognitive deficits in HD. Interestingly, increased NKCC1 appeared to occlude the effects of KCC2 reduction, suggesting that the reversal of GABA polarity was primarily due to increased NKCC1 rather than decreased KCC2 function. This finding is interesting, considering that Htt and KCC2 interact, and there is currently no evidence of any interaction between Htt and NKCC1. To conclude, the authors showed that daily intraperitoneal (IP) injections of the FDA-approved NKCC1 inhibitor, bumetanide, was sufficient to restore hippocampal-dependent memory in R6/2 mice. To confirm that the memory improvements were not due to off-target effects (Savardi et al., 2021), the authors used stereotaxic implantation of a micro-osmotic pump to deliver bumetanide to the brain, and found that this route of administration also restored cognitive deficits in R6/2 mice. Although bumetanide has proven to mitigate cognitive defects in the presymptomatic phase of HD, most patients are diagnosed with HD only after the onset of motor deficits and thus the HD-associated cognitive impairments produces a significant burden on caregivers and families (Domaradzki, 2015). Therefore, with the development of more sensitive methods for early diagnosis and the well-defined pattern of inheritance, treating premanifest HD patients by targeting CCCs appears to be a promising therapeutic strategy.

An obvious question is whether depolarizing GABA underlies motor deficits classically associated with HD. To address this, Hsu et al. (2019) demonstrated that NKCC1 transcripts and protein expression were higher in the striatum of the R6/2 and Hdh150q/7q mouse models and in the caudate nucleus of HD patients (Hsu et al., 2019). The authors demonstrated that the impairments in E_GABA_ progressively depolarized from ∼−60 mV to ∼−40 mV in 10-week-old to 16-week-old R6/2 mice, respectively. Of note, the authors did not discriminate between D1- and D2-MSNs in the striatum and since D2-MSNs are known to demonstrate synaptic dysfunction earlier, it is possible that the progressive depolarization in E_GABA_ was a result of the differential degeneration between the two pathways. In contrast to Dargaei et al. (2018), the authors attributed the aberrant CCC expression to neuroinflammation. The researchers rescued motor deficits by using an shRNA against NKCC1 in the striatum of R6/2 mice (shNKCC1) and that the expression of mHTT in astrocytes alone was sufficient to upregulate NKCC1. Determining whether the decrease in KCC2 expression happen before the increase in NKCC1 expression is important, as this will reveal prioritization for CCC-based therapeutic interventions.

In view of CCC dysfunction in HD and its importance for the maintenance of synaptic inhibition, we now discuss Cl^–^ regulation via CCCs in the basal ganglia, the primary site of histopathological damage in HD (Albin, 1995).

## Chloride Dynamics and Gamma-Amino Butyric Acid Signaling in the Basal Ganglia

### Overview

As explained above, chorea presents in earlier stages of the disease, while late stages are characterized by progressive hypokinesia and bradykinesia (Huntington, 1872). These biphasic changes throughout disease progression are caused by differential susceptibility in the direct and indirect pathways of BG circuitry. We first provide a brief overview of the BG, before delving into GABAergic signaling in this group of highly interconnected subcortical nuclei. The BG is primarily associated with motor control, including motor learning and fine-tuning motor behavior, and various non-motor behaviors, including emotional regulation, decision making and learning and memory (Graybiel et al., 1994; Graybiel and Grafton, 2015). Structures within the BG include the striatum, the Nucleus accumbens (NAcc), the internal (GPi) and external segments of the globus pallidus (GPe), the subthalamic nucleus (STN), substantia nigra pars reticulata (SNr) and substantia nigra pars compacta (SNc) (Alexander and Crutcher, 1990; Hedreen and DeLong, 1991). Input structures such as the striatum, NAcc, and STN, receive efferents from cortical, thalamic and nigral regions forming the beginnings of the direct, indirect and hyper-direct pathways (Lanciego et al., 2012). These input structures send their outputs to the intrinsic nuclei within the BG which include the GPe, STN and SNc, which then relay BG information between the input and output structures. Output structures include the GPi and the SNr, which receive information from other BG output nuclei and send projections to the ventral nuclei of the thalamus, which then sends BG information back to the cerebral cortex to form the cortico-BG-thalamocortical (CBGTC) loop (Alexander et al., 1986; Albin et al., 1989). Recently, as part of a consortium to fully explore the primary motor cortex, Foster et al. (2021) examined the CBGTC loop to map multi-synaptic output pathways of striatal domains (Foster et al., 2021), and determined that the direct pathway had greater convergence of striatal inputs than the indirect pathway, suggesting greater specificity of the indirect pathway. This decreased degree of informational convergence in the GPe could provide some insight on the susceptibility of the indirect pathway in HD.

### Chloride Dynamics and Gamma-Amino Butyric Acid Signaling in the Direct and Indirect Pathways

A recent study examined the effects of Cl^–^ dynamics on SNr responses to GABAergic pallidal and striatal inputs (activation via indirect and direct pathway, respectively) to ultimately make predictions about its impact on behavior (Phillips et al., 2020). SNr responses to GABAergic inputs from the direct and indirect pathway were determined to be diverse, with excitatory effects attributed to intracellular Cl^–^ handling. Stimulation of GPe and striatal projections resulted in biphasic inhibitory-to-excitatory responses in SNr neurons that were mediated by rapid Cl^–^ accumulation, which can overwhelm Cl^–^ extrusion via KCC2. Using optogenetic simulation, the researchers discovered that activation of soma-projecting GPe-neurons led to a greater proportion of excitatory responses in SNr−neurons, compared to dendritic-projecting striatal neurons, which suggests that the E_GABA_ in the soma is more depolarized than in the dendrites. This is somewhat surprising given that the difference in volume between the soma and dendrites (which affect the rates of Cl^–^ accumulation), predicts that the limited volume in dendrites should undergo faster rates of Cl^–^ accumulation, and therefore exhibit a more depolarized E_GABA_ (Doyon et al., 2016). However, this difference in E_GABA_ can be attributed to increased firing rate of GPe neurons compared to striatal neurons and/or the preferential localization of KCC2 in the dendrites of GABAergic SNr neurons compared to the soma (Gulácsi et al., 2003). Considering this work, it appears that high GPe GABAergic output can lead to increased Cl^–^ loading in the soma of SNr neurons, which can lead to greater/faster collapse of the Cl^–^ gradient through indirect pathway activation, consequently leading to excitatory GPe control of the SNr. This change in GABA polarity also compromises the ability of striatal neurons (mainly D1-MSNs of the direct pathway) to inhibit SNr dendrites, which may result in slow response times or even no response in perceptual decision-making tasks (Phillips et al., 2020). Interestingly, tonically spiking dopaminergic neurons in the SNr do not express KCC2, though it has been postulated that the weak GABAergic inhibitory drive is necessary for tonic release of dopamine (Gulácsi et al., 2003).

**FIGURE 3:**
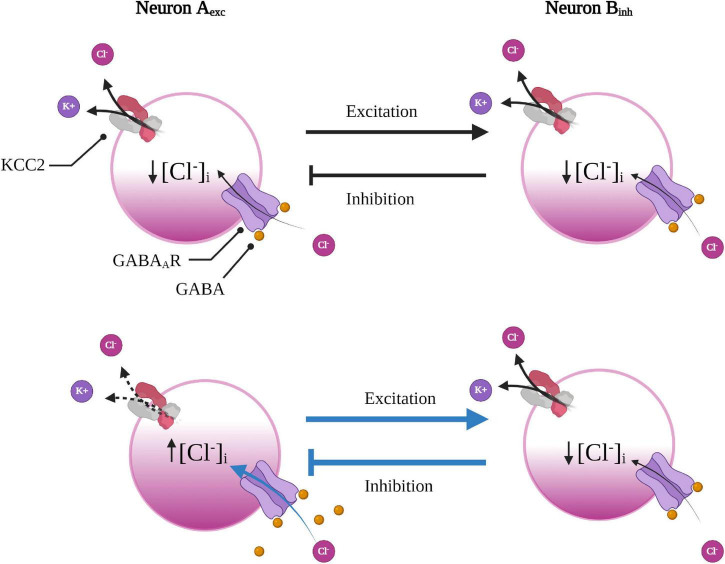
Underlying network connection can render some neurons more susceptible to GABAergic disinhibition. Excitatory neuron A is reciprocally connected with inhibitory neuron B to form a small feedback loop. With a reduction in KCC2 function (or increase in NKCC1, not shown here), in excitatory neuron A (below), Cl^–^ will accumulate leading to a faster rate of collapse in the Cl^–^ driving force subsequently weakening GABAergic inhibition. As neuron A experiences less inhibition, it will fire more, leading to greater excitation of the inhibitory neuron B. As a result of increased excitatory input, the inhibitory neuron will then increase inhibitory input back onto the excitatory neuron A, which will further enhance the Cl^–^ load in the excitatory neuron to create a deleterious loop. Created with BioRender.com.

As D2-MSNs degenerate earlier in the disease, striatal connectivity to the GPe is lost, leading to overactivation of GPe neurons, which is believed to underlie chorea (Figure 3; Crossman, 1987; Reiner et al., 1988). In human HD patients, deep brain stimulation of the GPe has proven beneficial in treating motor and cognitive dysfunction, although the mechanisms underlying this dysfunction remains unclear (Da Cunha et al., 2015; Wojtecki et al., 2016). However, in R6/2 mice, GPe neurons have proven to be hyperexcitable and blockade of GABA_A_Rs facilitated bursting activity (Akopian et al., 2016). Though the mechanisms leading to bursting are not fully understood, this finding leads us to hypothesize that KCC2 and/or Cl^–^ homeostasis is impaired in the GPe and/or other BG nuclei. Based on the predictions of this model, and if our hypothesis is correct (i.e., Cl^–^ handling is impaired in the GPe and/or other BG structures), the consequences can be far-reaching. With reduced KCC2 function and GPe hyperexcitability in HD, this could create a potentially deleterious loop within the BG circuitry, changing GABA polarity, particularly along the indirect pathway. Intuitively, with enhanced excitability, increasing inhibition appears to be the most logical answer. But as we consider GABA actions in the BG and the dynamic nature of Cl^–^ regulation, it becomes clear why enhancing GABAergic conductance can worsen neuronal dysfunction rather than temper it.

### Activity-Dependent Disinhibition Due to Cl^–^ Accumulation in the BG

**FIGURE 4:**
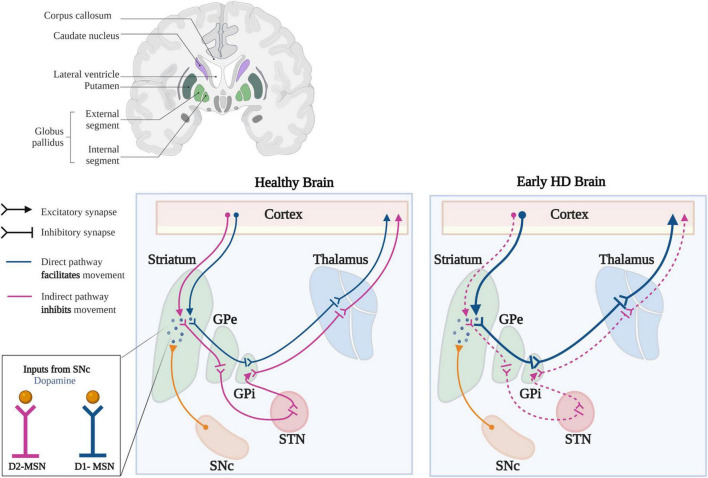
Synaptic changes in the basal ganglia circuitry in HD. In the healthy brain, activation of D1-MSNs of the direct pathway (blue) leads to disinhibition of the thalamus, which increases excitatory feedback to the motor cortex. Activation of the D2-MSNs of the indirect pathway (purple) leads to inhibition of the thalamus, which decreases excitatory feedback to the motor cortex. Dopaminergic inputs from the SNc produces different outcomes in D1- and D2-MSNs; inhibiting D2-MSNs, while exciting D1-MSNs. Early in HD, there is a preferential loss of D2-MSNs leading to increased output along the direct pathway, mediated by D1-MSNs. The decreased inhibitory output through the indirect pathway and increased output through the direct pathway, lead to the development of unwanted, hyperkinetic movements called chorea (GPe, Globus pallidus externa; GPi, Globus pallidus interna; STN, Subthalamic Nucleus; SNc, Substantia Nigra pars compacta). Template taken and modified with BioRender.com.

An important factor in determining the susceptibility to activity-dependent disinhibition is the underlying neural network topology (Doyon et al., 2011, 2016). To illustrate this point, one can imagine a simple reciprocal connection between two neurons that form a small feedback loop; whereby an inhibitory neuron provides negative feedback to a neuron from which it receives excitatory input. Reduced KCC2 function (or increased NKCC1) in the excitatory neuron would lead to impaired inhibition, and therefore would increase its firing rate. Increased firing would consequently lead to enhanced excitatory input onto the inhibitory neuron. As the inhibitory neuron experiences increased excitation, it would then increase inhibitory feedback onto the excitatory neuron, which would further enhance the Cl^–^ load in the excitatory neuron (Figure 4). This scenario provides insight as to why some networks, such as the BG, might be more vulnerable to activity-dependent disinhibition than others. As inhibition is required for fine-tuning gain and modulating oscillatory activity within neural networks (Doyon et al., 2016), a mild reduction in KCC2 function in one component might lead to catastrophic failure of feedback loops embedded in inhibitory networks. For example, abnormal oscillatory activity in the BG circuitry was hypothesized to underlie resting tremor in Parkinson’s disease (PD) (Plotkin and Goldberg, 2019). The theory that tremors were caused by altered rhythmogenesis within the CBGTC loop was due to the discovery that the STN and GPe were autonomous pacemakers that gave rise to spontaneous oscillatory activity (Plenz and Kital, 1999). The STN and GPe are reciprocally connected, forming a small subcircuit much like the scenario described above (Figure 3), where GPe neurons inhibits STN neurons, which also provides excitatory feedback to the GPe. Based on the predictions of our hypothetical scenario (Figure 3) and the known HD-associated dysfunction in the GPe and STN, one can imagine what might happen if the Cl^–^ regulation is overwhelmed in the GPe and/or the STN due to reduced KCC2 function. Indeed, the results become more consequential when considering that a portion of GPe neurons project back to the striatum, forming another (disinhibitory) feedback loop (Abdi et al., 2015). Due to the presence of these reciprocal and convergent connections, which form multiple closed-feedback loops, it seems impossible to pinpoint accurately where and when synaptic dysfunction might originate. Any structure within the indirect pathway might experience increased synaptic delays in negative-feedback due to dysregulated gain, leading to destabilization of the CBGTC loop (Plotkin and Goldberg, 2019). Based on the underlying network topology and the computational modeling in predicting the effects of dysregulated Cl^–^ in the BG, it is tempting to speculate whether this might play a role in the enhanced susceptibility of the indirect pathway due to impaired Cl^–^ handling.

## Chloride Dynamics and Gamma-Amino Butyric Acid Signaling in the Healthy and Huntington’s Disease Striatum

### Overview

In HD, striatal degeneration is highly systematic and undergoes extensive neuronal loss of MSNs while striatal interneurons are relatively spared (Vonsattel and DiFiglia, 1998). Although the preferential neurodegeneration of MSNs has been extensively studied in HD, the mechanisms remain unclear. MSN exhibiting low spontaneous activity (Plenz and Kitai, 1998), which may be due to strong feedforward inhibition from surrounding parvalbumin-positive (PV +) interneurons and/or intrinsic membrane properties that make it difficult for MSNs fire (Tepper and Plenz, 2004; Burke et al., 2017). For instance, MSNs are characterized by their hyperpolarized resting membrane potential (∼80 to −85 mV) and low membrane resistance (Wilson and Kawaguchi, 1996). This hyperpolarized membrane potential is one of two preferred MSN states - the “down” and “up” states. In the down state, MSNs rarely fire, in contrast to its presence in the up state (∼−50 mV), which sits closer to action potential threshold (−40 to −35 mV) and requires strong convergent glutamatergic input and possibly depolarizing GABAergic signaling (Plenz, 2003). With low firing rates and a hyperpolarized resting membrane potential, it appears that MSNs evolved to convey excitatory information with high signal to noise ratio, allowing only entry of relevant signals into the striatum.

Understanding GABA signaling in the striatum and the functional differences of D1- and D2-MSNs provides some insight into the enhanced susceptibility of D2-MSNs in HD, although despite the wealth of knowledge surrounding the two MSN subtypes, this has proven to be a difficult task, which we highlight below.

### Chloride Dynamics and Gamma-Amino Butyric Acid Signaling in the Healthy Striatum

Earlier studies of MSN-MSN monosynaptic transmission demonstrated that GABA is depolarizing at rest (E_GABA_ ∼60 mV; V_m_ ∼85 mV) (Wilson and Kawaguchi, 1996; Plenz, 2003), which is thought to facilitate transition into the upstate (Plenz, 2003). Notably, depolarizing GABA could act to dampen shunting effects of rectifying K^+^ channels on cortical glutamatergic transmission at rest (Wilson, 1992; Plenz, 2003). In addition, the effects of GABA change at more depolarized membrane potentials close to spiking threshold, where GABA can effectively delay action potential firing (Koós and Tepper, 1999). This temporal modulation of GABA on MSN firing could impact spike timing necessary for cortico-striatal plasticity and network activity (Plenz, 2003). As GABAergic signaling in MSNs appears to act through shunting inhibition (as opposed to hyperpolarizing inhibition), it’s important to note that this also relies on the Cl^–^ gradient and is susceptible to breakdown via Cl^–^ accumulation. Without restoration of the Cl^–^ driving force, shunting inhibition becomes ineffective, especially when modulating repetitive spiking. Although Cl^–^ flux can shunt excitatory input with an increase in membrane conductance, it also simultaneously decreases the membrane time constant leading to an increased firing rate, reducing the efficacy of shunting inhibition. This ineffectiveness in inhibitory signaling is especially prominent with either a depolarization in E_GABA_ or when GABA is depolarizing at rest such as in MSNs (Prescott et al., 2006).

In addition to lateral inhibition between neighboring MSNs, GABA signaling in the striatum modulates dopaminergic innervation and in turn modulates reinforcement learning (Cox and Witten, 2019). Activation of the D1 and D2 dopamine receptors produce functionally opposing outcomes, with D1 receptor activation exciting direct pathway MSNs, while D2 activation inhibits indirect pathway MSNs (Surmeier et al., 2007; Tecuapetla et al., 2009). Notably, dopaminergic terminals projecting from the SNc and VTA also co-release GABA to inhibit striatal output (Tritsch et al., 2012). This finding was based on the timing of GABA conductance and IPSCs, which were observed to be significantly faster than would be expected if GABA were released by nearby interneurons; however, its possible that dopaminergic neurons activate GABA receptors indirectly. Striatal cholinergic interneurons (CINs) are another source of GABA, with roughly half of CINs demonstrating the ability to co-release acetylcholine and GABA (CGINs) (Lozovaya et al., 2018). In a model of PD, CGINs display impairments in excitation/inhibition balance due to dysregulated Cl^–^ levels, which could be rescued by pharmacological NKCC1 inhibition. Interestingly, in the zQ175^±^ mouse model of HD, CINs demonstrated enhanced GABAergic inhibition, mediated by D2-MSN collaterals and not D1-MSNs (Lim and Surmeier, 2020). Multiple sources of striatal GABA could potentially increase Cl^–^ accumulation and activity-dependent disinhibition in the striatum. Without a clear understanding of GABAergic neurophysiology in the healthy striatum, the challenge of understanding GABAergic dysregulation in the HD brain is even greater, as we explain below.

### Chloride Dynamics and Gamma-Amino Butyric Acid Signaling in the Huntington’s Disease Striatum

Alterations in GABA synapses have been demonstrated across a wide range of HD mouse models and corroborated in HD patients, which suggests reduced levels of striatal GABA (Perry et al., 1973; Spokes, 1980; Spokes et al., 1980). Because the impairments in GABA neurotransmission have already been extensively described [for review see references (Garret et al., 2018; Hsu et al., 2018)], we only highlight those impairments here. HD mouse models have demonstrated alterations in the subunit composition and distribution of GABA_A_Rs, resulting in impaired receptor kinetics and GABAergic tone (Hsu et al., 2018). Cortical and striatal neurons exhibit lower levels of GAD65 and GAD67 (Urquhart et al., 1975), and GABA is consistently decreased across multiple rodent models and from post-mortem brain samples from HD patients (Perry et al., 1973; Spokes et al., 1980; Reynolds and Pearson, 1987). In line with this, electrophysiological recordings from R6/2 and Q175 demonstrate altered GABA-mediated Cl^–^ currents in the striatum, cortex, and hippocampus (Cepeda et al., 2013; Wójtowicz et al., 2013; Hsu et al., 2018; Dargaei et al., 2019). GABA_A_R subunit composition in the striatum is significantly altered leading to aberrant kinetics of GABA_A_ currents, with differential expression of the α1 subunit, a key subunit for fast inhibitory synaptic currents (Vicini et al., 2001). The α2 subunit, a major component of GABA_A_Rs in striatal MSNs is also decreased in R6/1 mice, consistent with the decreased GABAergic current in MSNs (Du et al., 2017). Taken together, altered GABA_A_R subunit composition lead to complex changes in GABAergic neurotransmission, but whether these changes in striatal GABAergic inhibition result from direct or downstream effects of mHtt or whether they are a result of compensatory homeostatic mechanisms necessary to mitigate cortical hyperexcitability remains to be determined.

For instance, in presymptomatic mouse models of HD, cortical glutamatergic input to MSNs is dysregulated, exhibiting increased amplitude (Cepeda et al., 2003) and increased frequency of spontaneous excitatory events (André et al., 2011; Raymond et al., 2011). At later stages of HD, increased glutamatergic input is replaced by a reduction in excitatory synaptic activity in MSNs and is accompanied by alterations in synaptic markers such as synaptophysin and PSD-95 (Cepeda et al., 2003). These findings reflect an early enhanced excitability of cortical pyramidal neurons (CPNs) and/or impairments in presynaptic inhibition of CPNs demonstrating early pathologically enhanced activity along the cortico-striatal pathway. While the CPN-MSN connection progressive deteriorates, there is considerable loss of dendritic spines on MSNs, which occur at around the same time overt behavioral impairments begin to manifest (Cepeda et al., 2003). Strikingly, alterations in striatal GABAergic inhibition also demonstrates a biphasic pattern that is highly variable between mouse models (Hsu et al., 2018). In symptomatic HD mice, the frequency of GABA_A_R-mediated events is increased, and inhibitory input is increased onto MSNs (Cepeda et al., 2004). Although, these changes would indicate a reduced output of MSNs along both the direct and indirect pathways, changes in glutamate, GABA and dopamine differentially affect the direct and indirect pathway output (André et al., 2011; Barry et al., 2018; Plotkin and Goldberg, 2019), making it difficult to accurately predict the changes in BG circuitry. Given that mHtt is expressed in higher levels in the cortex compared to the striatum, one might predict that these regions would exhibit similar electrophysiological dysfunction or degeneration. However, since opposite changes in these two brain regions occur, it appears that neuronal dysfunction is not only mediated by a cell-autonomous mechanism (Creus-Muncunill and Ehrlich, 2019), but as previously discussed, the underlying network configuration may play a key role in the type of neuronal dysfunction.

### D1- and D2-MSNs Differential Vulnerability in Huntington’s Disease

Although it is well accepted that D2-MSNs are affected earlier than D1-MSNs in HD, the mechanisms underlying this enhanced vulnerability remains unclear. Physiological and morphological differences between D1- and D2-MSNs are thought to underlie the preferential degeneration of D2-MSNs. For example, D1-MSNs have an increased number of primary dendrites and greater extension of arborization, compared to D2-MSNs. These differences may contribute to different electrophysiological properties as D1-MSNs are known to have more hyperpolarized resting membrane potentials, a greater rheobase (Gertler et al., 2008), and lower intrinsic excitability (Willett et al., 2019). Moreover, D2-MSNs receive more cortical glutamatergic inputs, which also form larger synapses compared to those that synapse with D1-MSNs (Lei et al., 2004; Francelle et al., 2014). Taken together, these differences could render D2-MSNs more prone to excitotoxic damage, a hallmark feature in the mechanisms underlying neuronal dysfunction in HD (Cepeda et al., 2007; Cepeda and Levine, 2020). Despite the degeneration of D2-MSNs, some full-length mouse models of HD exhibit increased glutamatergic transmission in D1-MSNs earlier compared to D2-MSNs (André et al., 2011) and in the zQ175 knock-in model, D1-MSNs displayed more prominent morphological and electrophysiological changes (Goodliffe et al., 2018). This suggests that D1-MSNs experience increased sensitivity to the toxic effects of mHtt compared to D2-MSNs, although it is possible that D1-MSNs employ D1-specific intrinsic factors and/or mechanisms of compensation to provide some level of protection.

Selective vulnerability of D2-MSNs may also be a result of differential dopaminergic signaling. D2-MSNs display reduced phosphorylation of glycogen synthase kinase-3 (GSK3), which has been proposed to play a neuroprotective role HD (Fernández-Nogales et al., 2015). In contrast, pathologically increased GSK3 was reported to contribute to the pathogenesis of HD and inhibiting GSK3 has proven to be neuroprotective (Carmichael et al., 2002). The conflicting evidence regarding the involvement of GSK3 in HD likely stem from the opposing actions of the different GSK3 isoforms (D’Mello, 2021). GS3Kβ phosphorylates Thr906-KCC2 and KCC2 activity (Watanabe et al., 2019), and inhibition of GSK3β enhances KCC2 expression and restores E_GABA_ in a model of nerve-injury pain (Yeo et al., 2021). The potential effects of altered GSK3 activity on KCC2 activity in HD remains to be determined but offers an interesting and important avenue for exploration.

Transcriptomic analyses of single-nuclear RNA sequencing in striatal MSNs revealed differential responses of D1- and D2-MSNs to mHtt toxicity (Lee et al., 2020). For example, D2-MSNs exhibited an upregulation in the neurotrophin signaling pathway and a downregulation in mitochondrial oxidative phosphorylation (OXPHOS) mRNA followed by release of mitochondrial RNA, which occurred later in D1-MSNs. Although the BDNF receptor *Ntrk2* was downregulated in both D1- and D2-MSNs, only D2-MSNs exhibited an upregulation of *Bdnf* itself, which the authors suggested may be a D2-MSN-specific homeostatic response. Altogether, these functional differences between D1- and D2-MSNs calls for more targeted therapeutic strategies to combat cell-type specific excitotoxicity early on in D2-MSNs. The reasons for preferential neurodegeneration of D2-MSNs in HD and whether CCCs are dysregulated in both MSN subtypes remain elusive. Nonetheless, several potential mechanisms have yet to be addressed, which we discuss further.

## Potential Mechanisms of Cation-Chloride Cotransporter Dysfunction in Huntington’s Disease

### Altered Protein-Protein Interaction Between K^+^-Cl^–^ Co-transporter-2 and mHtt

**FIGURE 5:**
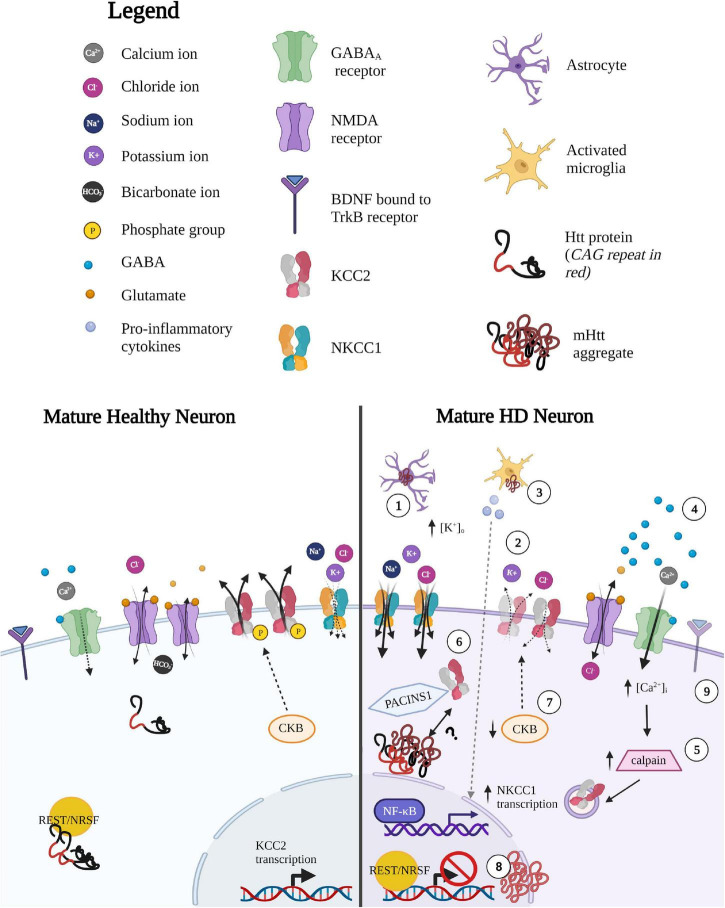
Potential mechanisms underlying altered CCC expression and function in HD. *(1) Impaired K* + *homeostasis in astrocytes leads to local increases in extracellular [K^+^.* Since Cl^–^ extrusion via KCC2 is driven by the K^+^ concentration gradient, small changes in extracellular K^+^ concentration (such as during sustained neuronal activity or neuronal excitability due increases in extracellular K^+^), can therefore *(2)* lead to reversal in the direction of ion transport to mitigate neuronal K^+^-mediated excitability. (3) *Inflammatory-mediated upregulation of NKCC1 expression.* NKCC1 transcripts are upregulated by the release of pro-inflammatory mediators released by activated microglia such as pro-inflammatory cytokine tumor necrosis factor alpha (TNF-alpha), which mediated by the nuclear factor kappa-light-chain-enhancer of activated B cells (NF-κB) pathway. *(4) Glutamate-mediated excitotoxicity leads to calpain-mediated degradation of KCC2.* Enhanced glutamate signaling leads to NMDA-mediated excitotoxic effects, resulting in increased Ca^2+^ influx. *(5)* Calcium-dependent protease, calpain, is well-studied in HD and is a strong negative regulator of KCC2 expression via cleavage of the C-terminal tail of KCC2 to reduce KCC2 expression. *(6) Altered interaction between KCC2 and (m)Htt.* Htt and KCC2 interaction is altered in either a gain-of-function or loss-of-function manner. The former would lead to greater/tighter binding of mHtt and KCC2 and as mHtt forms cellular aggregates, enhanced binding would result in reduced KCC2 from the surface membrane to reduce KCC2 activity. mHtt sequestering KCC2 may occur indirectly, via protein kinase C and casein kinase II substrate in neurons (PACSIN1). PACSIN1 is known to be sequestered in mHtt aggregates and also forms strong interactions with KCC2, pulling KCC2 from the membrane to restrict surface expression. Alternatively, Htt may play some role in stabilizing KCC2 in the membrane, a function that may be lost with mHTT protein. (7) *Reduced KCC2 function due to decrease in Brain-type creatine kinase (CKB)* activity. CKB is an important player in regenerating of ATP through the phosphocreatine-creatine kinase system. The downregulation of CKB is well described as mHtt suppresses the activity of the promoter of the CKB gene, lowering somatic CKB expression in HD mice and human patients. CKB is a strong positive modulator of KCC2 activity, therefore KCC2 and Cl^–^ dysregulation could be a result of the progressive downregulation of CKB in HD. CKB may enhance KCC2 in two ways: (i) CKB regenerates ATP required to regenerate the K^+^ gradients that drive KCC2 Cl^–^ extrusion (via Na^+^/K^+^ ATPase) and (ii) CKB is known to phosphorylate KCC2 through its capacity to autophosphorylate. *(8) Reduced transcription of KCC2 via pathological entry of REST/NRSF into the nucleus.* Normal Htt binds to REST/NRSF from binding and reducing transcripts containing DNA repressor element (RE1) also referred to as neuron-restrictive silencer element (NRSE) sequences, which is recognized by the transcriptional silencer RE-1 silencing transcription factor (REST), also known as neuronal restrictive silencing factor, (NRSF). Normal Htt promotes transcription of RE-1-containing transcripts by sequestering REST/NRSF in the cytoplasm. In contrast, mHtt allows entry of REST/NRSF into the nucleus, which may reduce KCC2 transcripts. *(9) Altered BDNF-TrkB signaling leads to altered KCC2 activity.* The regulatory effects of BDNF on KCC2 expression and function are dependent on activity and developmental stage. BDNF-TrkB signaling in immature neurons leads to the upregulation of KCC2 via ERK1/2 signaling, while in mature neurons BDNF-TrkB- mediated activation of the Ras/MAPK pathways leads to downregulation of KCC2. The underlying mechanism between BDNF-TrkB signaling on KCC2 expression in the context of HD remain unclear, however, it may be possible that there is a reversion to a neuronal immature state in order to “reboot” the system, leading to different regulatory effects of BDNF-TrkB on KCC2. Created with BioRender.com.

Based on the unbiased Htt transcriptome studies and the findings from Dargaei et al. (2018), it remains unclear how the HD mutation leads to CCC dysfunction (Dargaei et al., 2018). Given that Htt and KCC2 interact (either directly or indirectly), it is unclear whether this interaction is altered in a gain-of-function or loss-of-function manner. The former would promote the interaction between mHtt and KCC2, and as mHtt forms cellular aggregates, enhanced binding would reduce KCC2 in the surface membrane, thereby reducing KCC2 activity. Along the same lines, mHtt sequestering KCC2 may also occur indirectly, via PACSIN1, a predominant interactor of KCC2, which could pull KCC2 from the membrane to restrict its surface expression (Figure 5; Mahadevan et al., 2017). Alternatively, it is possible that Htt could stabilize KCC2 in the membrane, a function that may be lost with mHtt much like the reduced interaction of mHtt and REST/NSRF in the regulation of BDNF transcription. If the former scenario is true, and mHtt aggregate formation leads to reduced KCC2 membrane expression, it remains to be determined whether this is true across all rodent models without the presence of nuclear aggregates or whether this occurs very early on before these aggregates are even formed (Menalled and Chesselet, 2002). Regardless, a depolarizing shift in E_GABA_ may not necessitate aggregate formation, as the reversal of GABA polarity in HD is primarily due increased NKCC1 activity compared to decreased KCC2 function (Dargaei et al., 2018), despite there being no known interaction between NKCC1 and (m)Htt. Given that the changes in NKCC1 expression are present in presymptomatic HD mice, and in light of the novel neurodevelopmental emergence of the disease previously described, determining whether the “developmental” switch in GABA polarity even occurs is a possibility that warrants further examination.

### Post-translational Modulators of K^+^-Cl^–^ Co-transporter-2 Expression Are Altered in Huntington’s Disease: CKB and Calpain

Creatine kinases (CKs) are important players in energy homeostasis, particularly for the regeneration of ATP through the phosphocreatine-creatine kinase system (Allen, 2012). The downregulation of Brain-type creatine kinase (CKB) is well described in HD mouse models and human patients (Mochel et al., 2012; Lin et al., 2013), where mHtt is known to lower somatic CKB expression in primary neurons and striatum of HD mice (Lin et al., 2013). In the striatum of R6/2 mice, CKB protein expression is downregulated during the pre-symptomatic phase, however, the CKB transcript only decreases during late stages of the disease. A creatine-supplemented diet is sufficient to increase expression of CKB, reduce aggregate formation (Lin et al., 2013), and improve motor dysfunction and hearing impairments in R6/2 mice (Ferrante et al., 2000). Given that CKB is a strong positive modulator of KCC2 activity, where suppression of CKB activity leads to reduced KCC2 function (Inoue et al., 2006), it is possible that the depolarization of E_GABA_ and Cl^–^ dysregulation could be a result of the progressive downregulation of CKB in HD (Figure 5). The positive modulation of CKB on KCC2 happens in two ways: (i) CKB regenerates ATP necessary to regenerate the K^+^ gradients that drive KCC2 Cl^–^ extrusion (via Na^+^/K^+^ ATPase) and (ii) CKB phosphorylates KCC2 to increase activity (Hemmer et al., 1995; Inoue et al., 2004). Therefore, the beneficial effects of CKB in HD may also indirectly enhance KCC2, a possibility worth exploring.

KCC2 activity is also regulated by calpain, a calcium-dependent protease that has been implicated in many neurological diseases such as traumatic brain injury, neuropathic pain, and epilepsy (Ono et al., 2016; Lam et al., 2017). Calpain activation is well-studied in HD, as it plays a role in cleavage of Htt in the caudate of HD patients (Gafni and Ellerby, 2002), and inhibition of calpain is known to reduce toxicity (Gafni et al., 2004). The negative effects of calpain on KCC2 are well-documented and are a common cause of disinhibition in neurodegenerative disorders, as NMDAR-mediated excitotoxicity results in calpain-mediated cleavage of the C-term tail of KCC2 (Figure 5; Lee et al., 2011; Puskarjov et al., 2012). During seizure onset, m-calpain is excessively activated and KCC2 expression can be restored with a calpain inhibitor (Wan et al., 2018). Interestingly, seizures are observed in ∼40% of early onset HD patients but are relatively rare in adult-onset HD patients (Cloud et al., 2012). This phenomenon is also observed in mouse models, where R6/2 mice display increased susceptibility to seizures, although it is rare in full-length models with more protracted development of symptoms (Ferrante, 2009). Whether the beneficial effects of calpain inhibition also indirectly act on KCC2 surface stability (along with Htt levels) in HD remain to be determined.

### Reduction of BDNF in Huntington’s Disease and Its Impact on K^+^-Cl^–^ Co-transporter-2 Expression

As previously outlined, Htt functions to positively regulate BDNF transcription by preventing REST/NRSF from binding and reducing *bdnf* transcripts (Zuccato et al., 2003). Based on this regulatory function of Htt, it has been postulated that Htt acts as a general regulator of transcription RE1/NRSEs-containing genes through the interaction with REST/NRSF. One such transcript is *SLC12A5*, which contains two RE-1 sites; one located in the 5′ promoter region of KCC2b, which acts in concert with intronic RE-1 to repress the KCC2b transcripts (Yeo et al., 2009). Therefore, it is reasonable to suggest that due to the loss-of-function of mHTT, pathological entry of REST/NRSF into the nucleus may also reduce KCC2 transcription.

The regulatory effects of BDNF on KCC2 expression and function are complex and depend on both activity and developmental stage. In immature neurons, BDNF-TrkB signaling leads to the upregulation of KCC2 in an activity-dependent manner via ERK1/2 signaling (Uvarov et al., 2006), while in mature neurons, BDNF activation of TrkB receptors downregulates KCC2 surface expression in an activity-dependent manner (Rivera et al., 2002, 2004). This BDNF-TrkB mediated downregulation of KCC2 is well-characterized and underlies impaired KCC2 activity in many neuropathologies (Liu et al., 2019a; Pradhan et al., 2019), which lends strength to the hypothesis that alterations in BDNF signaling underlie KCC2 dysfunction in HD. This hypothesis is supported by experimentation where bath application of BDNF reduced the frequency of spontaneous GABAergic synaptic currents in R6/2 MSNs, further strengthening the link between BDNF and synaptic inhibition (Cepeda et al., 2004). Notably, TrkB expression is higher in D2-MSNs compared to D1-MSNs, which may lead to more pronounced effects of aberrant BDNF-TrkB signaling in D2-MSNs (Baydyuk and Xu, 2014). However, as outlined above, only D2-MSNs exhibited an upregulation of *Bdnf*, even though TrkB transcripts were downregulated in both D1 and D2-MSNs (Lee et al., 2020), which could potentially lead to even greater KCC2 dysfunction in the already vulnerable MSN subtype. Taken together, the increased baseline TrkB expression, enhanced HD-associated *Bdnf* increase and its implications on KCC2 function, could provide some insight into how KCC2 and Cl^–^ regulation may collapse more readily in D2-MSNs. Although, these finding present a logical yet puzzling assumption: with a loss of BDNF in HD, KCC2 levels should be left unchecked, therefore enhancing KCC2 function. Considering the complex pathways and context-dependent effects BDNF-TrkB signaling has on KCC2 expression, it is difficult to tease apart in HD. However, in many neurological disorders, a reversion to a neuronal immature state to “reboot” the system, might lead to different regulatory effects of BDNF-TrkB on KCC2, that have yet to be explored.

### Inflammatory-Mediated Upregulation of Na^+^-K^+^-Cl^–^ Co-transporter-1 Expression

The transcriptional upregulation of NKCC1 by cytokines and inflammation has been previously described, such as during pulmonary edema (Weidenfeld and Kuebler, 2017). As previously outlined, cytokine-mediated upregulation of NKCC1 transcription and protein expression have been demonstrated in the striatum of HD mice, leading to motor deficits (Figure 5; Hsu et al., 2019). Here, the authors validated cytokine-mediated upregulation of NKCC1 expression by incubating STHdH97 cells with the pro-inflammatory cytokine tumor necrosis factor alpha (TNF-alpha), mediated by the nuclear factor kappa-light-chain-enhancer of activated B cells (NF-κB) pathway. In HD, inflammation is most prominent during the final stages, suggesting that upregulation of NKCC1 may occur at later stages, though CCC changes were already evident in presymptomatic R6/2 mice (Dargaei et al., 2018). Activation of the D2 receptors (and not D1 receptors) can activate PP2A and inhibit the production of inflammatory cytokines and chemokines (Han et al., 2017); because PP2A can dephosphorylate Ser940 on KCC2 (Lee, 2009), it is possible that enhanced dopamine signaling early in HD (Chen et al., 2013) leads to an early PP2A-mediated reduction of KCC2 activity specifically on D2-MSNs. The activation of PP2A via D2 receptor activation would then produce two important effects on Cl^–^ regulation: (i) KCC2 downregulation via Ser940 dephosphorylation via PP2A (Lee et al., 2011) and/or (ii) the reduction of inflammatory cytokines production (Han et al., 2017), which could reduce NKCC1 transcripts. Therefore, it may be possible that reduced KCC2 function may be due to D2 receptor-mediated upregulation of PP2A or that it is a by-product in an attempt reduce inflammatory cytokine production.

### Reversal of Chloride Transport Through Increased K^+^ Extracellular Concentration

So far, the focus on KCC2 dysfunction has been on Cl^–^ dynamics and its deleterious effects on synaptic inhibition, but as KCC2 cotransports K^+^ and Cl^–^, K^+^ regulation inextricably becomes dysregulated. KCC2 extrudes Cl^–^ through a secondary active transport mechanism and is driven by the K^+^ concentration gradient. Thus, the direction of Cl^–^ transport can be reversed by even small changes in extracellular K^+^ concentration, which can occur during sustained neuronal activity to buffer extracellular K^+^ (Thompson and Gähwiler, 1989; Payne, 1997; Chamma et al., 2012). Therefore, under high stimulation and without KCC2 to re-establish Cl^–^ gradients, the local build up of K^+^ extracellular would further accelerate the progressive weakening of GABAergic currents. In HD, striatal MSNs have elevated levels of extracellular K^+^, due to abnormal astrocyte-mediated K^+^ homeostasis via impaired Kir4.1 channels, leading to neuronal excitability (Figure 5; Tong et al., 2014), which could lead to a reversal in the direction of ion transport via KCC2 to mitigate neuronal K^+^-mediated excitability.

## Cation-Chloride Cotransporters as Therapeutic Targets

### Overview

Although the alterations in GABA neurotransmission are well characterized in HD, drugs that target GABA signaling have proven to be limited. For instance, clinical trials using synthetic agonists of δ- containing GABA_A_Rs (Foster et al., 1983) and the GABA analog, muscimol (Shoulson et al., 1977) failed to improve motor or cognitive function in HD patients. However, when we consider CCCs as the root cause of impaired inhibition in HD, these results may not be surprising. Increasing the GABAergic conductance would still impose a Cl^–^ load on neurons which could exacerbate synaptic dysfunction (Prescott et al., 2006). Therefore, the goal should be directed to restoring Cl^–^ homeostasis and thereby inhibition rather than *increasing* GABAergic transmission, which would be counterproductive without mechanisms to re-establish the Cl^–^ gradient, as we outline below.

### Targeting Na^+^-K^+^-Cl^–^ Co-transporter-1

The FDA-approved diuretic, bumetanide, has proven to be beneficial in rescuing various symptoms of many neurological disorders for decades (Puskarjov et al., 2014; Ben-Ari, 2017). Bumetanide blocks NKCC1 activity at low doses to restore physiological intracellular Cl^–^ levels and GABAergic inhibition, and has been successful in treating core symptoms in epilepsy, various models of autism, schizophrenia, pain, PD, Down syndrome, and brain trauma (Ben-Ari, 2017). As bumetanide has been used for decades, the side-effects have been well characterized (Ben-Ari, 2017), and are due to off-target effects on the peripherally expressed NKCC2, which is found in the kidney (Igarashi et al., 1996). Moreover, due to bumetanide’s systemic administration, low brain-plasma ratio of bumetanide due to low blood-brain barrier permeability have cast doubt on bumetanide’s ability to act on the brain (Römermann et al., 2017). To address these concerns, a novel, selective and safe NKCC1 inhibitor called ARN23746 was recently discovered (Savardi et al., 2020). ARN23746 is metabolically stable, exhibits high solubility and could restore core symptoms in mouse models of Down syndrome and autism. More importantly, ARN23746 displayed no off-target effects and no toxicity with chronic treatment *in vivo*. Thus, the development of selective chemical inhibitors such as ARN2376 continue to allow key players in Cl^–^ regulation to remain promising druggable targets.

### Targeting K^+^-Cl^–^ Co-transporter-2

K^+^-Cl^–^ co-transporter-2 is a prime target for restoring Cl^–^ homeostasis due to the reduced likelihood of developing adverse side effects as it is neuron-specific, operates under isotonic conditions and does not contribute to volume regulation (Kahle et al., 2008). In addition, KCC2 operates near its equilibrium point (Buzsáki et al., 2007) and increasing Cl^–^ extrusion capacity beyond physiological levels is unlikely as the ability of inhibition to reach pathological levels are restricted by the non-linear relationship between KCC2 and E_GABA_ (Doyon et al., 2011, 2016) and is constrained by E_K_^+^ (Düsterwald et al., 2018). However, one might consider the possibility that enhanced KCC2 may lead to pathological accumulation of extracellular K^+^, which could increase neuronal excitability (Kaila et al., 1997). However, large shifts in the driving force of Cl^–^ due to enhanced KCC2 activity exhibited only subtle changes in volume (<1%) and KCC2 expression proved to have very little correlation with the neuronal membrane potential (Düsterwald et al., 2018). Since excess neuronal firing is the primary culprit of increased extracellular K^+^ (and hence pathological neuronal excitability) (Doyon et al., 2011, 2016), enhancing KCC2 function to decrease excess firing provides further support for KCC2 as a promising therapeutic target.

To enhance KCC2 function, the CLP series (CLP265, −290, −657) of Cl^–^ extrusion enhancers have been developed; they are reported to enhance KCC2 membrane expression, lower intracellular Cl^–^ levels and restore core symptoms in rodent models of neuropathic pain (Gagnon et al., 2013). CLP drugs have since then been extended to treat a wide range of diseases but despite their effectiveness, they have a few caveats. The timing of administration is critical, and the mechanism of action is not fully known, which has been suggested to act on GABA_A_Rs (versus KCC2) (Cardarelli et al., 2017). Despite this, CLP’s proven effectiveness and increasing use in a variety of diseased models offers hope for the use of KCC2 as a continued therapeutic target.

Recently, post-translational regulators have been an alternative approach to enhance KCC2 activity (Zhang et al., 2020). For instance, using an inhibitor of the master CCC regulator SPAK kinase (STK39), called ZT-1a, researchers were able to mitigate cerebral infarction in a model of ischemic stroke. Targeting SPAK kinases is a powerful way of regulating Cl^–^ as it can simultaneously inhibit NKCC1 and activate KCC2 (Huang et al., 2019), thus proving to be an effective approach to modulate CCCs for various neurological disorders.

## Conclusion

K^+^-Cl^–^ Co-transporter-2 and NKCC1 regulate steady-state intracellular Cl^–^ gradients and consequently, the direction and strength of GABAergic transmission. During neuropathological insult, neurons revert to a phenotypically immature state with increased Cl^–^ levels, leading to depolarizing effects of GABA. Several lines of evidence presented here, demonstrate an important link between CCC dysfunction and HD (Dargaei et al., 2018; Hsu et al., 2019), though the mechanisms underlying this dysfunction remain unclear. HD is a multi-factorial disease, with no effective treatments or cure and sadly, many of the current therapeutic strategies are limited by the late detection of the disease. As outlined here, CCC-targeted therapies have proven to ameliorate the cognitive and motor impairments associated with HD in both presymptomatic and symptomatic mouse models, providing a novel therapeutic strategy.

In this review, we summarized key pieces of experimental evidence that underscores the importance of Cl^–^ regulation and GABA signaling in the healthy and diseased BG neural circuit and how the underlying network topology may render the BG susceptible to activity-dependent disinhibition. Based on this knowledge, it becomes clear why drugs used to enhance GABA conductance have failed to improve HD symptoms in clinical trials. We also present possible mechanisms that may underlie CCC dysfunction, although given the complexity of the disease, further investigation is necessary to uncover the exact mechanisms. Given the developmental defects present in HD and the importance of CCC during neurodevelopment, future experimental work during various developmental stages is needed to explore this possibility to uncover novel therapeutic avenues.

## Author Contributions

MS drafted the review and created the figures. MW revised and finalized the review. Both authors contributed to the article and approved the submitted version.

## Conflict of Interest

The authors declare that the research was conducted in the absence of any commercial or financial relationships that could be construed as a potential conflict of interest.

## Publisher’s Note

All claims expressed in this article are solely those of the authors and do not necessarily represent those of their affiliated organizations, or those of the publisher, the editors and the reviewers. Any product that may be evaluated in this article, or claim that may be made by its manufacturer, is not guaranteed or endorsed by the publisher.
