# Corrigendum: Striatal Chloride Dysregulation and Impaired GABAergic Signaling Due to Cation-Chloride Cotransporter Dysfunction in Huntington's Disease

**DOI:** 10.3389/fncel.2022.876821

**Published:** 2022-03-11

**Authors:** Melissa Serranilla, Melanie A. Woodin

**Affiliations:** Department of Cell and Systems Biology, University of Toronto, Toronto, ON, Canada

**Keywords:** KCC2, chloride regulation, GABA, synaptic inhibition, striatum, Huntington's disease

In the original article, there was a mistake in the legend for **Figures 3, 4** as published. The legends for those figures were swapped. The corrected legends appear below.

**FIGURE 3 |** Underlying network connection can render some neurons more susceptible to GABAergic disinhibition. Excitatory neuron A is reciprocally connected with inhibitory neuron B to form a small feedback loop. With a reduction in KCC2 function (or increase in NKCC1, not shown here), in excitatory neuron A (below), Cl^−^ will accumulate leading to a faster rate of collapse in the Cl^−^ driving force subsequently weakening GABAergic inhibition. As neuron A experiences less inhibition, it will fire more, leading to greater excitation of the inhibitory neuron B. As a result of increased excitatory input, the inhibitory neuron will then increase inhibitory input back onto the excitatory neuron A, which will further enhance the Cl^−^ load in the excitatory neuron to create a deleterious loop. Created with BioRender.com.

**FIGURE 4 |** Synaptic changes in the basal ganglia circuitry in HD. In the healthy brain, activation of D1-MSNs of the direct pathway (blue) leads to disinhibition of the thalamus, which increases excitatory feedback to the motor cortex. Activation of the D2-MSNs of the indirect pathway (purple) leads to inhibition of the thalamus, which decreases excitatory feedback to the motor cortex. Dopaminergic inputs from the SNc produces different outcomes in D1- and D2- MSNs; inhibiting D2-MSNs, while exciting D1-MSNs. Early in HD, there is a preferential loss of D2-MSNs leading to increased output along the direct pathway, mediated by D1- MSNs. The decreased inhibitory output through the indirect pathway and increased output through the direct pathway, lead to the development of unwanted, hyperkinetic movements called chorea (GPe, Globus pallidus externa; GPi, Globus pallidus interna; STN, Subthalamic Nucleus; SNc, Substantia Nigra pars compacta). Template taken and modified with BioRender.com.

The authors apologize for this error and state that this does not change the scientific conclusions of the article in any way. The original article has been updated.

## Publisher's Note

All claims expressed in this article are solely those of the authors and do not necessarily represent those of their affiliated organizations, or those of the publisher, the editors and the reviewers. Any product that may be evaluated in this article, or claim that may be made by its manufacturer, is not guaranteed or endorsed by the publisher.

